# The Importance of Metabolic and Environmental Factors in the Occurrence of Oxidative Stress during Pregnancy

**DOI:** 10.3390/ijms241511964

**Published:** 2023-07-26

**Authors:** Miljana Z. Jovandaric, Sandra Babic, Misela Raus, Biljana Medjo

**Affiliations:** 1 Department of Neonatology, Clinic for Gynecology and Obstetrics, University Clinical Center of Serbia, 11000 Belgrade, Serbia; 2 Department of Gynecology and Obstetrics, Clinic for Gynecology and Obstetrics, University Clinical Center of Serbia, 11000 Belgrade, Serbia; 3 Department of Neonatology, University Children’s Hospital, 11000 Belgrade, Serbia; 4 Faculty of Medicine, University of Belgrade, 11000 Belgrade, Serbia; 5 Department Pediatrics and Neonatal Intensive Care, University Children’s Hospital, 11000 Belgrade, Serbia

**Keywords:** pregnancy, lipid metabolism, oxidative stress, diseases

## Abstract

Metabolic changes in pregnant women begin in the first weeks after conception under the influence of placental hormones that affect the metabolism of all nutrients. An increased concentration of total lipids accompanies pregnancy and an increased accumulation of triglycerides in low-density lipoproteins (LDL) particles. Lipids in small dense LDL particles are more susceptible to oxidative modification than normal-density LDL particles. Unlike LDL high-density lipoproteins (HDL), lipoprotein particles have an atheroprotective role in lipid metabolism. The very growth of the fetus depends on the nutrition of both parents, so obesity is not only in the mother but also in the father. Nutritional programming of the offspring occurs through changes in lipid metabolism and leads to an increased risk for cardiometabolic diseases. Pregnancy is accompanied by an increased need for oxygen in the mitochondria of the placenta and a tendency to develop oxidative stress. Oxidative stress represents a disturbance in the balance of oxidation–reduction processes in the body that occurs due to the excessive production of free oxygen radicals that cellular homeostatic mechanisms are unable to neutralize. When the balance with the antioxidant system is disturbed, which happens when free oxygen radicals are in high concentrations, serious damage to biological molecules occurs, resulting in a series of pathophysiological and pathological changes, including cell death. Therefore, oxidative stress plays a significant role in the pathogenesis of many complications that can occur during pregnancy. The oxidative status of pregnant women is also influenced by socioeconomic living conditions, lifestyle habits, diet, smoking, and exposure to environmental air pollution. During a healthy pregnancy, the altered lipid profile and oxidative stress create an increased risk for premature birth and pregnancy-related diseases, and a predisposition to adult diseases.

## 1. Introduction

Pregnancy is a dynamic process of a woman’s organism at the local systemic level, which is accompanied by numerous metabolic changes in order to adapt to the needs of placenta formation, rapid fetal development, childbirth, and lactation [1]. Changes in the metabolic state vary among pregnant women depending on lifestyle habits, health status before and during pregnancy, and fetal growth dynamics. Nutrients such as glucose, free fatty acids, unsaturated fatty acids, amino acids, vitamins, and minerals are needed for normal fetal development [2]. The growth of the fetus is influenced by the eating habits of not only the mother but also the father. Obesity in men and women exerts consequences on offspring through nutritional programming. Nutritional programming of offspring occurs through changes in lipid metabolism and leads to an increased risk for cardiometabolic diseases [3,4].

Metabolic changes in pregnant women occur in early pregnancy. In the first weeks after conception, the placenta is formed, which begins to secrete hormones that affect the metabolism of all nutrients. Human chorionic gonadotropin (hCG) is detected in serum and urine a few days after implantation; it is produced by trophoblast cells that surround the growing embryo (initially syncytiotrophoblast), which forms the placenta after implantation. Its concentrations increase during early pregnancy and reach a maximum during the first two months after conception, after which they decrease, maintaining low values until the delivery date. Its role is immunomodulatory; it is reflected in the attraction of Treg in the uterus and the mother’s tolerance towards the recognition of the fetus [5].

The balance between Treg and Th17 cells is necessary to maintain the normal course of pregnancy, while a shift in the ratio of Th17/Treg to Th17 cells is a potential cause of pregnancy complications, including preeclampsia, recurrent spontaneous abortion, and gestational diabetes mellitus [6,7]. Decidual immune cells (DICs) not only regulate the maternal immune response to promote fetal hemiallograft tolerance but also mediate trophoblast implantation and invasion. At the same time, trophoblasts mediate fetal–maternal interactions for the exchange of nutrients, gases, and waste products, and for the regulation of immune tolerance [8]. Human placental lactogen (hPL) is a polypeptide hormone produced by placental syncytiotrophoblast cells. hPL increases progressively during pregnancy, and its full function is not fully elucidated [9].

Since it is biologically similar to growth hormone, it is possible that it represents a type of growth factor for the fetus and placenta. In addition, it has a significant metabolic role in the metabolism of carbohydrates and lipids, and the availability of nutrients to the fetus [10]. The placenta is the primary source of steroid hormones, including the synthesis of estrogen and progesterone [11]. Estrogens and progesterone, in addition to their effects on the reproductive organs, play a role in the regulation of insulin and glucose homeostasis, lipid metabolism, and appetite regulation, which can be important in promoting metabolic changes in the mother during pregnancy. In addition, progesterone and estrogen have opposite effects on food intake [12].

Estrogen reduces food intake partly by inducing the production of leptin in adipose tissue, while progesterone increases food intake by increasing the level of neuropeptide Y (NPY) and reducing the expression of the peptide cocaine and amphetamine-regulated transcript (CART) by the hypothalamus [13,14]. The course of early pregnancy can be called the anabolic phase, while the course of late pregnancy is accompanied by complex metabolic processes owing to the interaction of mother and fetus and is designated as the catabolic phase [15].

Fatty acids participate in the synthesis of cell membranes and are also an energy source. During the first weeks of pregnancy, cholesterol is transferred to the placenta with the help of LDL cholesterol, from where it is later released into the fetal circulation. Cholesterol participates in the synthesis of steroid hormones and cell membranes. During the first weeks of pregnancy, serum triglycerides are 20% higher, and by the end of pregnancy, they reach concentrations that are three times higher compared to nonpregnant women. Other serum lipids (cholesterol, phospholipids, fatty acids) also increase during pregnancy, but to a lesser extent. Fat accumulation occurs as a result of increased sensitivity to insulin and an increased number of insulin receptors in adipose tissue, and thus increased activity of tissue lipoprotein lipase [16].

Placental hormones such as placental growth hormone, human placental lactogen, leptin, and tumor necrosis factor-α (TNF-α), which are responsible for insulin resistance, are thought to play a role in fat accumulation during pregnancy. The constant increase in estradiol during pregnancy contributes to the increase in lipid concentration so that most women develop a lipid profile by the end of pregnancy that can be considered atherogenic in healthy nonpregnant women [17].

Embryonic and later fetal growth and development are very sensitive to the intrauterine environment. Unfavorable conditions in the intrauterine environment create a predisposition for the development of diseases, from the development of prenatal structural defects to metabolic diseases after birth through mitochondrial dysfunction, oxidative stress, and protein modification [18]. A changed lipid profile during pregnancy can be the basis for the development of metabolic and cardiovascular diseases in the child [19].

## 2. Metabolic Changes in Pregnancy

### Lipid Metabolism in Pregnancy

Lipid metabolism is an important factor in fetal development, the course, and the outcome of pregnancy. In healthy pregnant women, triglyceride values increase two to four times, while the increase in cholesterol is about 25–50%. However, the mechanism leading to these adaptive changes has not been fully elucidated [20].

Pregnancy is accompanied by an increase in LDL cholesterol and free fatty acids. The value of HDL cholesterol increases in the second trimester and maintains the achieved value until the end of pregnancy. These changes during pregnancy represent a necessary adaptation in the pregnant woman’s metabolism to the demands of the fetus and prepare the pregnant woman for the upcoming birth and lactation [21].

An increase in the value of triglycerides in the plasma corresponds to an increase in the value of VLDL lipoprotein particles. Through cholesteryl ester transfer protein (CETP), whose activity increases in the middle of pregnancy, there is an exchange of triglycerides between VLDL and LDL or HDL particles, so that there is an increased accumulation of triglycerides in these particles as well [22].

During pregnancy, the activity of hepatic lipase (HL) decreases, which enables the conversion of HDL-2 particles into HDL-3 lipoprotein particles, which leads to the accumulation of HDL-2 particles rich in triglycerides [23]. The physiological increase in lipids plays an essential role during pregnancy, which is reflected in the mobilization of free fatty acids from fat depots in late pregnancy, which is necessary for fetal growth and steroid synthesis in the placental tissue [24,25].

Free fatty acids are necessary for the development of the fetal brain and visual system, and, as biosynthetic precursors for hormones, cross the placenta by oxalic diffusion [26]. Fatty acids are first converted to acyl-CoA. Acyl-CoA can then be esterified into various lipids, triglycerides, phospholipids, and cholesterol esters. For the passage of fatty acids to the fetus, the role of the placenta is played by the expression of genes for placental lipase enzymes, which include lipoprotein lipase (LPL), endothelial lipase (EL), and hormone-sensitive lipase (HSL), which release fatty acids from acyl-CoA, triglycerides, and phospholipids [27].

In addition, the placenta has a large number of receptors for binding VLDL particles. In the catabolic phase, placental hormones increase the production of VLDL particles and decrease the activity of hepatic lipase, which, together with the increased expression of VLDL̸apoE receptors in the placenta, enables the appropriate influx of triglyceride-rich lipoproteins of the mother into the fetoplacental unit [28]. Insufficient supply of lipids to the fetus, which is associated with disturbed fetoplacental influx associated with an abnormal lipid profile, and which is reflected in reduced values of total cholesterol, triglycerides, LDL, and HDL-cholesterol, may be the cause of intrauterine fetal growth retardation (IUGR) [29].

LDL cholesterol contains seven subfractions, i.e., particles that differ in size, density, chemical composition, and function in the body. LDL_1–2_ is a subfraction of larger particles that are antiatherogenic, while the fraction of smaller and denser LDL_3–7_ particles, more sensitive to oxidative modification compared to LDL particles of normal density, show greater binding to proteoglycans of the vessel wall, and reduced uptake by LDL receptors [30]. Highly atherogenic small dense lipoproteins LDL_3–7_ (sdLDL) dominate in 97% of women in the 35th and 36th weeks of pregnancy. Small dense LDL_3–7_ particles are also thought to contribute to endothelial dysfunction in preeclampsia [31]. Considering the specifically changed lipid profile during pregnancy, the question arises whether hyperlipidemia during pregnancy is dyslipidemia. Classic dyslipidemia is characterized by a decrease in HDL cholesterol, which is not the case during an uncomplicated pregnancy. In preeclampsia, this feature of classic dyslipidemia with a decrease in HDL cholesterol is seen [32].

Triglycerides are esters of glycerol and fatty acids. Esterification is carried out with one, two, or all three hydroxyl groups of glycerol with fatty acids, whereby mono, di, or triglycerides are formed. Their basic role in the body consists in the creation of energy depots from which fatty acids are released as needed, and their oxidation provides the energy necessary for the life of all cells. The largest amount of triglycerides is found in the composition of fatty tissue (about 95%), while small amounts are present in the blood. Even for triglycerides, it is now reliably established that their increased amount in the blood plays a significant role in the process of atherosclerosis [33]. During pregnancy, the TG/HDL-chole index is particularly interesting, given the manifest hypertriglyceridemia during pregnancy. High TG can be complicated by low HDL-C. Although both parameters are mutually independent risk factors for ASCVD, the most relevant cases are those with simultaneous high TG and low HDL-C, which has a strong atherogenic effect. It has been proven that a ratio (TG/HDL-chole) over 0.5 is considered an indicator of atherogenicity [34,35]. In pregnant women, VLDL levels increase due to decreased activity of LPL and hepatic lipase. During pregnancy, VLDL production is also facilitated by hormone-sensitive lipase in adipose tissue [36].

The increase in the value of VLDL particles during pregnancy is considered to be due to increased synthesis of these particles, and not compositional changes within the particles, considering that the VLDL/Chol index does not change significantly during pregnancy and is lower in all trimesters of pregnancy compared to nonpregnant women. Therefore, although the lipid metabolism in pregnancy is specifically changed, taking into account the lipid and lipoprotein indices, it is balanced hyperlipidemia [37].

Studies have shown that there is a so-called difference between triglycerides and LDL subfractions “threshold” effect. Namely, with low and normal triglyceride values, there is a positive correlation with the LDL-2 subfraction, and with triglyceride values above 1.5 mmol/L, there is a positive correlation with the LDL-3 subfraction, which is relatively constant below the stated triglyceride values. In the first trimester of pregnancy, the increased value of triglycerides may be responsible for the increase in small dense LDL particles [38].

The total cholesterol in the fetal plasma is proportionally increased with the cholesterol values of the pregnant woman during the second trimester of pregnancy and slowly decreases with the growth of the pregnancy, that is, the gestational age of the fetus. It has been proven that lipid concentrations in the umbilical blood of fetuses of healthy pregnant women are significantly lower than lipids in the blood of pregnant women, and elevated HDL cholesterol and a lower LDL-chole/HDL-chole ratio in umbilical blood indicate that the fetus is protected from lipid atherogenicity during a healthy pregnancy. On the other hand, it is believed that hypercholesterolemia during pregnancy, although limited and transient, can cause the development of pathological changes and fatty streaks in the aorta of the fetus, thereby influencing the development of atherosclerosis in the offspring’s later life [39].

The reason for fetal fat accumulation may be gender differences despite similar placental exposure to excess TG or FFA. Total body fat at birth is higher in female newborns from 38 to 42 gestational weeks compared to the amount of body fat in male newborns. The reason for gender differences in the amount of fat in newborns is the placental reaction to the same environment depending on the gender of the fetus. Epigenetic mechanisms that regulate placental differentiation and function, and thus fetal growth and phenotype, may have contributed to this programming process. The metabolic status of the mother can change the expression and function of the placental gene with direct consequences on the development of the fetal tissue, i.e., the influence on the phenotype of the offspring [40]. During the second trimester of an uncomplicated pregnancy, there is an increase in HDL cholesterol value and this value is maintained until the delivery date, which is primarily due to the effect of estrogen. The antiatherogenic effect of HDL cholesterol is explained by its functional protection, mediating the transport of cholesterol from peripheral tissues to the liver, but also by its anti-inflammatory and antioxidant role. Concerning HDL cholesterol subfractions, we usually describe two fractions: HDL-2, which is larger, and HDL-3, which is smaller and denser and has a lower atheroprotective character [41].

**Figure 1:**
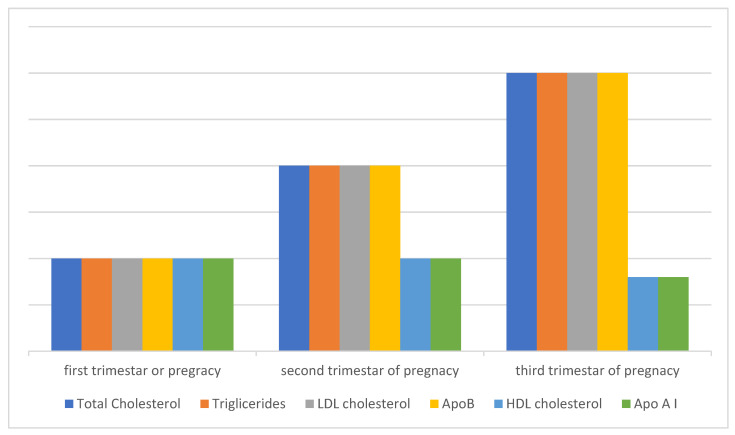
Lipid metabolism during a healthy pregnancy. Total cholesterol and triglycerides increase significantly already in early pregnancy and continue to increase during pregnancy, so that by the end of pregnancy cholesterol increases by about 30% and triglycerides almost three times. The values of LDL and HDL cholesterol also change during pregnancy, while the values of apolipoprotein AI (ApoAI) and apolipoprotein B (ApoB) during pregnancy generally follow the values of the lipoprotein particles in which they are composed, ApoAI in the composition of HDLa and ApoB in the composition of LDLa. A specific characteristic of this altered lipid profile during physiological pregnancy is an increase in the value of HDL cholesterol, which has an atheroprotective effect. This characteristic distinguishes hyperlipidemia of physiological pregnancy from pathological dyslipidemia, which is responsible for the onset of atherosclerosis towards the end of pregnancy, when the proatherogenic components (cholesterol, triglycerides, ApoB, LDL) continue to increase, while the HDL value does not increase but remains close to the level of the second trimester, even with the tendency for a slight decrease in value, and this may also mean its reduced antioxidant and anti-inflammatory functionality, which further points to different mechanisms responsible for the development of atherosclerosis. The fact that HDL increases during pregnancy and reaches a maximum in the second trimester suggests the possibility of other adaptive and protective mechanisms involved in late pregnancy. After childbirth, the changed lipid profile is maintained for a long period, which can be up to a year or more. The value of HDL in the cumulative effects of multiple pregnancies remains permanently lower, which also represents a risk for developing cardiovascular diseases in a woman’s later life.

It is believed that HDL is also subject to lipid peroxidation, even more easily than LDL, and since it is a smaller molecule than LDL, it diffuses more easily into the subendothelial space. When it comes to HDL subfractions, the HDL-2 fraction is more susceptible to oxidation than the HDL-3 fraction [42]. An altered lipid profile during pregnancy has the characteristics of a proatherogenic lipid profile and can affect the development of cardiovascular disease (CVD) in a woman’s later life. Certain studies have shown a positive correlation between the number of pregnancies and the risk of developing CVD in later life, which can be explained by lower HDL cholesterol values in later life [43]. Also, studies have shown that women with a greater number of pregnancies have a higher risk of developing diabetes mellitus later in life [44] (Figure 1).

## 3. Oxidative Stress in Pregnancy

Pregnancy is a state of increased sensitivity to oxidative stress, which occurs mainly due to the increased need for oxygen in the mitochondria of the placenta. Full-term pregnancy involves a series of events, including embryogenesis, embryo implantation, fetoplacental development, fetal growth, and delivery. Pregnancy increases oxidative stress, a phenomenon that arises from the normal systemic inflammatory response, resulting in large amounts of circulating reactive oxygen species (ROS), produced by the placenta [45].

Oxidative stress during pregnancy can lead to cell damage. In situations where oxidative stress overcomes the antioxidant defense of the placenta, tissue damage occurs [46]. Oxidative stress represents a disturbance in the balance of oxidation–reduction processes in the body that occurs due to the excessive production of free oxygen radicals (ROS), which cellular homeostatic mechanisms are unable to neutralize. Free oxygen radicals play a significant role as secondary messengers in many cellular signaling processes, and their high reactivity originates from one or more unpaired electrons. When the balance with the antioxidant system is disturbed, which happens when free oxygen radicals are in high concentrations, serious damage to biological molecules occurs, resulting in a series of pathophysiological and pathological changes, including cell death. Therefore, oxidative stress plays a significant role in the pathogenesis of many complications that may occur during pregnancy that may affect fetal development [47].

The high reactivity of molecular oxygen enables its participation in high-energy processes that take place in cells containing mitochondria, where the process of oxidative phosphorylation takes place [48].

Over 90% of oxygen from the air in the body is reduced to water by receiving four electrons from the electron transport system in the mitochondrial respiratory chain [49].

Molecular oxygen in this system is used to obtain water, and the successively released energy during electron transport is used to obtain electrochemical potential and synthesis of ATP. Electron transport is a stepwise process that starts with hydrogen, i.e., coenzyme dehydrogenase, and ends with the cytochrome c oxidase enzyme complex [50].

The mitochondrial electron transport system is one of the most important processes in which the superoxide anion radical is generated. Molecular oxygen, diffusely present in cells, is tightly bound to the cytochrome c oxidase enzyme complex. However, since this bond on the electron transporters that are in the respiratory chain before the cytochrome c oxidase system is not so tight, some of the transported electrons can be transferred to molecular oxygen, forming ROS [51,52].

Superoxide anion can also be formed within the shorter electron transport chain within the endoplasmic reticulum during protein synthesis and biotransformation of exogenous and endogenous substrates [53]. Other sources of superoxide under physiological conditions include NADPH oxidase, cytochrome P450, and other oxidoreductases. Various growth factors, drugs, toxins, inflammation, UV exposure, and lipid peroxidation, can also lead to increased ROS generation. The enzyme xanthine dehydrogenase under normal conditions breaks down purines, xanthine, and hypoxanthine to uric acid, using NAD+ as an electron acceptor. In the state of hypoxia, the formation of hypoxanthine increases with the transfer of electrons to molecular oxygen and the formation of superoxide anions [54].

Superoxide is neutralized in the presence of the enzyme superoxide dismutase (SOD), whereby hydrogen peroxide (H_2_O_2_) is formed, which is less reactive and is not a free radical, but since it is involved in the creation and detoxification of free radicals, it is considered part of ROS. Hydrogen peroxide is degraded to water by the action of catalase and glutathione peroxidase and is considered an oxygen metabolite in aerobic cellular metabolism [55]. H_2_O_2_ occurs in normal mammalian cellular metabolism and is an important metabolite in oxidative stress. The term “oxidative eustress” denotes physiological oxidative stress, as opposed to excessive load, “oxidative distress”, which causes oxidative cellular damage [56].

In a damaged antioxidant environment, with superoxide anion, and in the presence of iron, Fe^2+^ forms hydroxyl ion (OH•) in Fenton’s reaction. The hydroxyl ion is highly reactive and its half-life is about 10–9 s. Due to its high reactivity, it reacts with every biological molecule found in its immediate vicinity, and also, due to the same high reactivity, the acceptor of this free radical is not known. The uncontrolled generation of superoxide also leads to a reaction with nitrogen monoxide (NO•) and the formation of peroxynitrite (ONOO−) and hypochlorous acid (HOCl) [57,58].

### 3.1. Causes of Oxidative Stress in Pregnancy

Oxidative status during pregnancy is also influenced by the lifestyle habits of pregnant women, such as smoking, physical activity, and exposure to environmental pollution, and socioeconomic living conditions. It is known that smoking during pregnancy, whether active or passive, can lead to a series of complications, such as spontaneous abortion, placental abruption, premature birth, intrauterine growth retardation of the fetus, or the low body weight of the newborn [59].

Cigarette smoke contains a mixture of compounds in a gaseous state or condensed in the form of tar, which are mostly oxidants and pro-oxidants capable of causing oxidative stress by increasing the generation of free radicals and depleting the antioxidant capacity of pregnant women. In addition, carbon monoxide and nicotine cross the placental barrier and cause uteroplacental insufficiency, thereby reducing the supply of nutrients and oxygen to the fetus [60]. Carbon monoxide binds to hemoglobin and forms carboxyhemoglobin, causing fetal hypoxia. Pregnant women who smoke give birth to children who have an average of 150–300 g less body weight compared to children of pregnant women who do not smoke, and smoking during pregnancy can also affect the postnatal growth and development of the child [61].

For normal outcome of pregnancy, healthy development of the fetus, and to maintain the health of the woman, an optimal body weight during pregnancy is certainly important. Pregnant women with a body mass index BMI > 30 kg m^−2^ are considered obese [62]. Women who have a prepregnancy body mass index greater than 25 kg m^−2^ have problems with conception and a higher risk of miscarriage and stillbirth, while obese women are more likely to develop complications during pregnancy, including preeclampsia and gestational diabetes. Also, research has shown that the value of BMI before pregnancy is a significant predictor for the development of preeclampsia. Preeclampsia and the development of cardiovascular disease in later life share common risk factors—endothelial dysfunction, oxidative stress, and increased inflammatory activity [63].

The view that overweight pregnant women and obesity are independently associated with oxidative stress is relatively new and, together with placental oxidative stress and lipid peroxidation, may contribute to the development of pregnancy complications. Oxidative stress can significantly change the processes important for the proper growth and development of the fetus, which is more or less sensitive during intrauterine development [64].

It is also known that increased oxidative stress during pregnancy leads to reduced body weight of the newborn, which is associated with an increased risk for the development of some of the chronic diseases in the later life of an adult, such as hypertension, diabetes, hypercholesterolemia, and cardiovascular diseases [65].

The Western way of eating is characterized by a high intake of saturated and omega-6 fatty acids, reduced intake of omega-3 fats, excessive use of salt, and too much sugar. However, substantial evidence from epidemiological and clinical studies has shown that Western dietary patterns (WDP), due to high intake of red meat, processed meat, refined grain products, sweets, fast food, and french fries, are associated with an increased risk of diseases such as type 2 diabetes, obesity, metabolic syndrome, and coronary heart disease [66]. Trans-fatty acids, which are formed during the frying process by polymerization, oxidation, and hydrogenation, lead to insulin resistance (IR) and an increased risk of developing gestational diabetes mellitus (GDM) and type 2 diabetes [67]. Frequent intake of red meat, which contains animal fats such as cholesterol and saturated fatty acids, and high protein intake can increase the risk of developing GDM [68]. In addition to the aforementioned meat components, heme iron from red meat and high plasma iron concentrations can promote oxidative stress through the Fenton reaction and increase the formation of hydroxyl radicals, which can cause IR or even damage pancreatic β-cells and reduce pancreatic insulin secretion over time. Processed meats are treated with nitrites and nitrates that react with amino compounds to form nitrosamines that can cause IR by affecting insulin receptor expression, inflammation, and increasing levels of oxidative stress [69].

With global urbanization and rapid industrialization, the problem of air pollution is becoming increasingly significant, affecting all regions and all age groups. Some scientists warn that air pollutants can increase the risk of respiratory and cardiovascular diseases by causing oxidative stress and DNA methylation, i.e., disruption of methylome reprogramming during early embryogenesis, which leads to physiological and metabolic changes in the fetus and altered susceptibility of the offspring to various diseases in later life [70].

The most sensitive population group to air pollution is pregnant women. The impact of air pollution on the course of pregnancy itself, the period of pregnancy, and the fetus has not yet been clarified [71]. Depending on the concentration and size of the particles in the air, the effects depend on the course of pregnancy and the development of the fetus. It is considered that the exposure of pregnant women to PM2.5 during pregnancy can increase the risk of low birth weight (TLBW) and the occurrence of hypothyroidism in newborns [72]. Exposure to PM2.5 greater than 13.8 mg/m^3^ during pregnancy results in a decrease in birth weight [73].

Depending on the emission of different components in the air during pregnancy, a decrease in birth weight may occur if the pregnant woman was exposed to high concentrations of SO_2_, while exposure to NO_2_ leads to fetal macrosomia [74]. Studies on the exposure of the fetus to air pollution and the effect on certain periods of pregnancy are still not unified. There is a lack of assessment of exposure in different trimesters of pregnancy, and the mechanisms by which air pollution affects the growth and development of the fetus [75]. Exposure to air pollution during late pregnancy can cause inflammation and, by causing hypoperfusion of the placenta, lead to preterm birth (PTB) [76].

Previous experiments on rats have revealed that artificial exposure of animals to PM2.5 particles causes increased levels of interleukin (IL)-1, IL-6, and tumor necrosis factor α (TNFα). Therefore, chronic inflammation with a disturbed concentration of cytokines and the presence of a huge amount of reactive oxygen species (ROS) has a role in the development of many diseases, including hypothyroidism. Exposure of female rats to PM2.5 particles reduce circulating thyroid hormone levels by interrupting thyroid hormone biosynthesis, biotransformation, and transport; by inducing oxidative stress and inflammatory responses that deregulate thyroid hormone secretion; by reducing serum FT4 levels; and by increasing the incidence of hypothyroidism [77]. Ionizing radiation during pregnancy causes changes in DNA and indirect damage, including the creation of hydroxyl radicals and hydrogen radicals. DNA damage implies the loss of bases or their damage, or the breaking of bonds between bases. The damage is most lethal to the cell and depends on the time and length of exposure. Incomplete cellular damage is subject to repair attempts that may be successful or lead to abnormal function [78].

**Figure 2:**
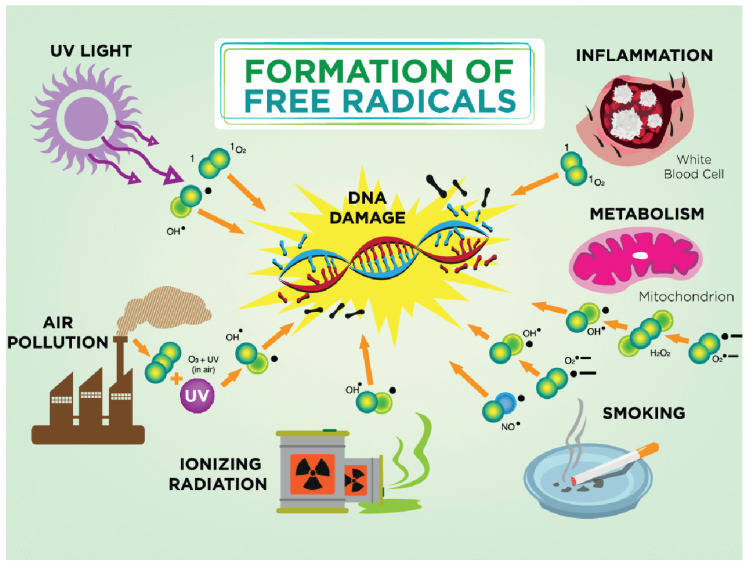
Formation of free radicals. In a damaged antioxidant environment, hydroxyl ion (OH•) is formed. The hydroxyl ion is highly reactive and its half-life is about 10–9 s. Due to its high reactivity, it reacts with every biological molecule that is in its immediate vicinity. The most common causes for free radicals in pregnancy are smoking, air pollution, ultraviolet radiation, and ionizing radiation; physiologically altered metabolism during pregnancy can lead to mitochondrial damage and DNA damage.

The goat is more or less exposed to ultraviolet rays every day. The consequences depend on the length of exposure and the type of ultraviolet radiation. Melanin is a skin pigment that is produced through the enzymatic oxidation of tyrosine and has antioxidant properties. Oxidative DNA damage in skin cells is most often caused by ROS obtained by photosensitization mediated by solar ultraviolet radiation (UVR) when the oxidation product 8-oxo-7,8-dihydro-2′-deoxyguanosine (8-oxodG) is formed. This product is incorporated with adenine and causes DNA mutations that play a role in carcinogenesis [79] (Figure 2).

### 3.2. Free Radicals

Radicals are normal components of cells, and they are formed by the action of oxygen molecules on cell membrane proteins. Under physiological conditions, most of the oxygen (about 80%) in the mitochondria of cells is reduced by cytochrome oxidase (without the formation of free radicals). The remaining 10–20% enters into further oxidation–reduction reactions in the cytoplasm and mitochondria where the oxygen superoxide anion radical (O^2−^) is formed. Free radicals (SR) are effectively neutralized by a series of enzymatic and nonenzymatic defense mechanisms that include superoxide dismutase, glutathione peroxidase, catalase, glutathione reductase, ascorbic acid, and tocopherol, and these act as free radical scavengers [80].

Free radicals are molecules or fragments of molecules with an unpaired electron in the outer orbit, which makes them highly reactive with a consequent affinity for binding to other molecules. In order to increase the stability of these molecules, there is a high probability of starting a series of reactions when there is peroxidation of lipid membranes and deoxyribonucleic acid (DNA) with consequent cell damage. Free radicals are unstable molecules, or ions of high reactivity, which in the body enter into chemical reactions with parts of the cell (proteins, lipids, carbohydrates, DNA molecules), thereby leading to biochemical, structural, and functional disorders. In a normal molecule, the nucleus is surrounded by a pair of negatively charged electrons. By removing one electron from the pair, through a process called oxidation, the molecule becomes unstable and destructive (a “radical” molecule is formed) [81]. Radicals react with neutral biological molecules, damaging them and creating new radicals (chain reaction). A radical can donate an electron to a nonradical and act as a reducing agent, creating new radicals. A radical can take an electron from a nonradical and thus become an oxidizing agent, again creating new radicals. A radical can “tear off” a hydrogen atom from a C-H bond in an organic molecule, creating “carbon centered” radicals (which with O_2_ make peroxyl radicals •RO_2_). In this way, DNA, cell membranes, and proteins are damaged. Radically induced lipid peroxidation reduces membrane fluidity, changes membrane transport, and damages membrane proteins, receptors, enzymes, and ions [82].

## 4. Consequences of the Action of Free Oxygen Radicals

### 4.1. Lipid Peroxidation

Lipid peroxidation occurs as a result of the action of free radicals on the polyunsaturated fatty acids of lipids, and to a lesser extent, it is present in all cells and tissues. Since lipids are an integral part of membranes, numerous cell organelles that contain membranes are exposed to damage caused by uncontrolled lipid peroxidation, which in turn puts into question not only the functioning of the cell but also its survival [83].

During the process of lipid peroxidation, the fluidity and permeability of the cell membrane change, the electrolyte transport through the membrane is disturbed, the electrical resistance decreases, and the mobility of membrane proteins occurs. Lipid peroxidation is considered the cause of numerous diseases such as atherosclerosis, diabetes, Parkinson’s disease, Alzheimer’s disease, and various chronic inflammatory conditions. It is also considered a cause of complications in pregnancy and is especially crucial in the development of preeclampsia [84].

Uncontrolled lipid peroxidation also leads to pathological changes during pregnancy in both the mother and fetus. The placenta plays a central role in fetal development, growth, communication between mother and fetus, homeostasis, and adaptation of pregnancy to injury, so placentas play an important role in pregnancy complications, such as fetal growth restriction, preeclampsia, premature birth, and abruption [85].

The mechanisms by which ferroptosis can play a role in placental dysfunction are that the placenta is normally susceptible to hypoxia–reoxygenation in early pregnancy, before as well as and during childbirth under the influence of uterine contractions; iron depots in placental trophoblasts are then actively transferred via the placenta to the developing fetus; and lastly, lower levels of glutathione peroxidase 4 (GPX4), a key enzyme that protects cells from the accumulation of harmful specific hydroxy-peroxidized phospholipids (Hp-PL) and ferroptosis, are associated with trophoblast damage, placental dysfunction, and the onset of preeclampsia [86].

### 4.2. Protein Damage

Although lipid peroxidation is at the center of research attention, protein oxidation, due to its large presence and numerous functions in the body, has been recognized recently as a possible main target for free radical action. The side chains of all amino acid residues of proteins are sensitive to oxidation caused by the action of ROS. Lipid peroxidation is associated with protein aggregation and leads to disruption of organelles and cellular functions. Maintaining the redox status of proteins is thought to be fundamental to maintaining normal cell function. Protein oxidation, and thus altered structure and function, and impaired homeostasis of plasma proteins, are associated with neurodegenerative diseases [87,88].

Highly reactive oxygen and nitrogen species (RONS) carry out lipid peroxidation of the cell membrane leading to intracellular accumulation of highly reactive molecules such as lipid peroxide (LOOH), malondialdehyde (MDA), 4-hydroxynonenal (HNE), and acrolein, which change the structure and thus the function of proteins [89]. Unsaturated fatty acids such as docosahexaenoic acid (DHA), eicosapentaenoic acid (EPA), and arachidonic acid (ARA) are most sensitive to the influence of RONS. Antioxidants can model lipid peroxidation in two ways, by directly scavenging RONS or indirectly by increasing and decreasing lipoxygenase enzymes [90].

### 4.3. DNA Damage

DNA damage is considered the most serious negative response of the cell to the excessive production of ROS and the oxidation of these molecules since it can cause mutations and genetic instability that are reflected in the outcome of pregnancy itself, whether it is complications during pregnancy or the quality and health of the fetus and newborn. Although the DNA repair system is very efficient, excessive production of ROS that leads to mitochondrial damage activates an inflammatory process that activates lymphocytes and macrophages, thus creating an inflammatory process with damage at the level of purine and pyrimidine bases. DNA oxidation is considered a significant factor in the development of carcinogenesis, diabetes, and aging [91] (Figure 2).

## 5. Prevention of Oxidative Stress during Pregnancy

Pregnancy is a dynamic process that involves systemic and local changes in the mother to support the supply of nutrients and oxygen to the fetus for growth in the womb. Disturbances in this process can lead to complications in pregnancy, changes in the trajectory of fetal growth, and premature birth. Maternal homeostasis is maintained by a variety of mediators including hormones, cytokines, oxidative status, and nutrient supply. Embryonic development occurs in a relatively low-oxygen environment and is highly sensitive to injury from oxidant molecules due to its low antioxidant capacity. Oxidative stress is associated with the creation of ROS, which has a physiological and pathological role in the placenta, embryo, and fetus [92]. Factors that disrupt this homeostasis, such as nutritional deficiency or excess, inflammation, oxidative stress, and lipotoxicity, can compromise fetal growth and development [93].

A well-balanced diet is the main source of nonenzymatic antioxidants. A healthy diet characterized by a high intake of fruits, vegetables, and whole grains, together with healthy fats, such as mono- and polyunsaturated fats, and a low intake of saturated fats are sources of bioactive compounds that act as components in antioxidant systems. Evidence from observational and interventional studies has shown that the Mediterranean diet (MD) can reduce concentrations of various biomarkers of oxidative stress and inflammation [94].

The use of the MD pattern was associated with lower levels of F2-isoprostanes and higher levels of total antioxidant capacity (TAC) in the adult population. In addition, the administration of MD led to significantly reduced levels of biomarkers reflecting various aspects of oxidative stress, such as biomarkers of lipid peroxidation (F2-isoprostanes) and oxidative DNA damage in adult subjects [95].

Adherence to healthy dietary patterns (i.e., a Mediterranean diet rich in antioxidants) during pregnancy is suggested to improve maternal health and facilitate proper fetal development by reducing levels of oxidative stress during a critical period of vulnerability; however, the relationship between maternal adherence to healthy eating patterns during pregnancy and biomarkers of oxidative stress in pregnant women and their offspring has not yet been proven [96].

## 6. Conclusions

All factors that act during a healthy pregnancy on the development of oxidative stress can lead to complications during pregnancy and later adult diseases.

## Data Availability

All data are available in the PubMed archives (MEDLINE database).

## References

[1] Wang J., Wu Z., Li D., Li N., Dindot S.V., Sattereld M.C., Bazer F.W., Wu G. (2012). Nutrition, epigenetics, and metabolic syndrome. Antioxid. Redox Sign..

[2] Tarry-Adkins J.L., Ozanne S.E. (2017). Nutrition in early life and age-associated diseases. Ageing Res. Rev..

[3] Cropley J.E., Eaton S.A., Aiken A., Young P.E., Giannoulatou E., Ho J.W., Buckland M.E., Keam S.P., Hutvagner G., Humphreys D.T. (2016). Male-lineage transmission of an acquired metabolic phenotype induced by grand-paternal obesity. Mol. Metab..

[4] Fernandez-Twinn D.S., Hjort L., Novakovic B., Ozanne S.E., Saffery R. (2019). Intrauterine programming of obesity and type 2 diabetes. Diabetologia.

[5] Wang S., Qian J., Sun F., Li M., Ye J., Li M., Du M., Li D. (2019). Bidirectional regulation between 1st trimester HTR8/SVneo trophoblast cells and in vitro differentiated Th17/Treg cells suggest a fetal-maternal regulatory loop in human pregnancy. Am. J. Reprod. Immunol..

[6] Lentz L.S., Stutz A.J., Meyer N., Schubert K., Karkossa I., von Bergen M., Zenclussen A.C., Schumacher A. (2022). Human chorionic gonadotropin promotes murine Treg cells and restricts pregnancy-harmful proin-flammatory Th17 responses. Front. Immunol..

[7] Qian J., Zhang N., Lin J., Wang C., Pan X., Chen L., Li D., Wang L. (2018). Distinct pattern of Th17/Treg cells in pregnant women with a history of unexplained recurrent spontaneous abortion. Biosci. Trends..

[8] Jing M., Chen X., Qiu H., He W., Zhou Y., Li D., Wang D., Jiao Y., Liu A. (2023). Insights into the immuno-modulatory regulation of matrix metalloproteinase at the maternal-fetal interface during early pregnancy and pregnancy-related diseases. Front. Immunol..

[9] Rassie K., Giri R., Joham A.E., Teede H., Mousa A. (2022). Human Placental Lactogen in Relation to Maternal Metabolic Health and Fetal Outcomes: A Systematic Review and Meta-Analysis. Int. J. Mol. Sci..

[10] Sibiak R., Gutaj P., Wender-Ozegowska E., Jankowski M., Mozdziak P., Kempisty B. (2020). Placental Lactogen as a Marker of Maternal Obesity, Diabetes, and Fetal Growth Abnormalities: Current Knowledge and Clinical Perspectives. J. Clin. Med..

[11] Costa M.A. (2016). The endocrine function of human placenta: An overview. Reprod. Biomed. Online.

[12] Napso T., Yong H.E.J., Lopez-Tello J., Sferruzzi-Perri A.N. (2018). The Role of Placental Hormones in Mediating Maternal Adaptations to Support Pregnancy and Lactation. Front. Physiol..

[13] Shende P., Desai D. (2019). Physiological and Therapeutic Roles of Neuropeptide Y on Biological Functions. Adv. Exp. Med. Biol..

[14] Yosten G.L., Haddock C.J., Harada C.M., Almeida-Pereira G., Kolar G.R., Stein L.M., Hayes M.R., Salvemini D., Samson W.K. (2021). Past, present and future of cocaine- and amphetamine-regulated transcript peptide. Physiol. Behav..

[15] Stern C., Schwarz S., Moser G., Cvitic S., Jantscher-Krenn E., Gauster M., Hiden U. (2021). Placental Endocrine Activity: Adaptation and Disruption of Maternal Glucose Metabolism in Pregnancy and the Influence of Fetal Sex. Int. J. Mol. Sci..

[16] Waage C.W., Mdala I., Stigum H., Jenum A.K., Birkeland K.I., Shakeel N., Michelsen T.M., Richardsen K.R., Sletner L. (2022). Lipid and lipoprotein concentrations during pregnancy and associations with ethnicity. BMC Pregnancy Childbirth.

[17] Kelly A.C., Powell T.L., Jansson T. (2020). Placental function in maternal obesity. Clin. Sci..

[18] Osredkar J. (2023). Impact of Endogenic and Exogenic Oxidative Stress Triggers on Pregnant Woman, Fetus, and Child. Int. J. Mol. Sci..

[19] Grilo L.F., Tocantins C., Diniz M.S., Gomes R.M., Oliveira P.J., Matafome P., Pereira S.P. (2021). Metabolic Dis-ease Programming: From Mitochondria to Epigenetics, Glucocorticoid Signalling and Beyond. Eur. J. Clin. Investig..

[20] Gootjes D.V., Posthumus A.G., Wols D.F., de Rijke Y.B., Van Lennep J.E.R., Steegers E.A.P. (2022). Maternal lipid profile in pregnancy and embryonic size: A population-based prospective cohort study. BMC Pregnancy Childbirth.

[21] Lippi G., Albiero A., Montagnana M., Salvagno G.L., Scevarolli S., Franchi M., Guidi G.C. (2007). Lipid and lipoprotein profile in physiological pregnancy. Clin. Lab..

[22] Roland M.C.P., Godang K., Aukrust P., Henriksen T., Lekva T. (2021). Low CETP activity and unique composi-tion of large VLDL and small HDL in women giving birth to small-for-gestational age infants. Sci. Rep..

[23] Loukidi-Bouchenak B., Lamri-Senhadji M., Merzouk S., Merzouk H., Belarbi B., Prost J., Belleville J., Bouchenak M. (2008). Serum lecithin:cholesterol acyltransferase activity, HDL2 and HDL3 composition in hypertensive mothers and their small for gestational age newborns. Eur. J. Pediatr..

[24] Chavan-Gautam P., Rani A., Freeman D.J. (2018). Distribution of Fatty Acids and Lipids During Pregnancy. Adv. Clin. Chem..

[25] Jovandaric M.Z., Ivanovski P.I. (2017). Free Fatty Acids of Newborns from Women with Gestational Diabetes Mellitus. Fetal Pediatr. Pathol..

[26] Perazzolo S., Hirschmugl B., Wadsack C., Desoye G., Lewis R.M., Sengers B.G. (2017). The influence of placental metabolism on fatty acid transfer to the fetus. J. Lipid. Res..

[27] Barrett H.L., Kubala M.H., Romero K.S., Denny K.J., Woodruff T.M., McIntyre H.D., Callaway L.K., Nitert M.D. (2014). Placental Lipases in Pregnancies Complicated by Gestational Diabetes Mellitus (GDM). PLoS ONE.

[28] Grace M.R., Vladutiu C.J., Nethery R.C., Siega-Riz A.M., Manuck T.A., Herring A.H., Savitz D., Thorp J.T. (2017). Lipoprotein particle concentration measured by nuclear magnetic resonance spectroscopy is associated with gestational age at delivery: A prospective cohort study. BJOG.

[29] Chassen S., Jansson T. (2020). Complex, coordinated and highly regulated changes in placental signaling and nutrient transport capacity in IUGR. Biochim. Biophys. Acta Mol. Basis Dis..

[30] Kopčeková J., Kolesárová A., Schwarzová M., Kováčik A., Mrázová J., Gažarová M., Lenártová P., Chlebo P., Kolesárová A. (2022). Phytonutrients of Bitter Apricot Seeds Modulate Human Lipid Profile and LDL Subfractions in Adults with Elevated Cholesterol Levels. Int. J. Environ. Res. Public Health.

[31] Ivanova E.A., Myasoedova V.A., Melnichenko A.A., Grechko A.V., Orekhov A.N. (2017). Small Dense Low-Density Lipoprotein as Biomarker for Atherosclerotic Diseases. Oxid. Med. Cell. Longev..

[32] Berberich A.J., Hegele R.A. (2022). A Modern Approach to Dyslipidemia. Endocr. Rev..

[33] Feitosa A.C.R., Barreto L.T., Silva I.M.D., Silva F.F.D., Feitosa G.S.F. (2017). Impact of the Use of Different Diagnostic Criteria in the Prevalence of Dyslipidemia in Pregnant Women. Arq. Bras. Cardiol..

[34] Tani S. (2020). The Ratio of Triglyceride to High-density Lipoprotein Cholesterol as an Indicator of Risk Stratification for Atherosclerotic Cardiovascular Disease in a Clinical Setting. Intern. Med..

[35] Moriyama K. (2020). The Association between the Triglyceride to High-density Lipoprotein Cholesterol Ratio and Low-density Lipoprotein Subclasses. Intern. Med..

[36] Cibickova L., Schovanek J., Karasek D. (2021). Changes in serum lipid levels during pregnancy in women with gestational diabetes: A narrative review. Biomed. Pap. Med. Fac. Palacky. Univ. Olomouc..

[37] Huang J.-K., Lee H.-C. (2022). Emerging Evidence of Pathological Roles of Very-Low-Density Lipoprotein (VLDL). Int. J. Mol. Sci..

[38] Wang Z., Peng Y., Mao S., Zhang L., Guo Y. (2023). The correlation between blood-lipid ratio in the first tri-mester and large-for-gestational-age infants. Lipids Health Dis..

[39] Mauri M., Calmarza P., Ibarretxe D. (2021). Dyslipemias and pregnancy, an update. Clin. Investig. Arterioscler..

[40] Barbour L.A., Hernandez T.L. (2018). Maternal Lipids and Fetal Overgrowth: Making Fat from Fat. Clin. Ther..

[41] Von Eckardstein A., Nordestgaard B.G., Remaley A.T., Catapano A.L. (2022). High-density lipoprotein revisited: Biological functions and clinical relevance. Eur. Heart J..

[42] Rohatgi A., Westerterp M., von Eckardstein A., Remaley A., Rye K.-A. (2021). HDL in the 21st Century: A Multifunctional Roadmap for Future HDL Research. Circulation.

[43] Aleksenko L., Quaye I.K. (2020). Pregnancy-induced Cardiovascular Pathologies: Importance of Structural Components and Lipids. Am. J. Med. Sci..

[44] Wang C.D., Ma Y., Li S.W., Li Q.X., Zhang L., Lin L.L., Huang B.B., Jiang Y.S. (2022). Analysis of risk factors for gestational diabetes mellitus in elderly multipara women in the next pregnancy. Zhonghua Yi Xue Za Zhi.

[45] Hardy M.L.M., Day M.L., Morris M.B. (2021). Redox Regulation and Oxidative Stress in Mammalian Oocytes and Embryos Developed In Vivo and In Vitro. Int. J. Environ. Res. Public Health.

[46] Hussain T., Murtaza G., Metwally E., Kalhoro D.H., Kalhoro M.S., Rahu B.A., Sahito R.G.A., Yin Y., Yang H., Chughtai M.I. (2021). The Role of Oxidative Stress and Antioxidant Balance in Pregnancy. Mediat. Inflamm..

[47] Sultana Z., Maiti K., Aitken J., Morris J., Dedman L., Smith R. (2017). Oxidative stress, placental age-ing-related pathologies and adverse pregnancy outcomes. Am. J. Reprod. Immunol..

[48] Pospíšil P., Prasad A., Rác M. (2019). Mechanism of the Formation of Electronically Excited Species by Oxidative Metabolic Processes: Role of Reactive Oxygen Species. Biomolecules.

[49] Kumar R., Jafri M.S. (2022). Computational Modeling of Mitochondria to Understand the Dynamics of Oxidative Stress. Methods Mol. Biol..

[50] Yin Y., Shen H. (2022). Common methods in mitochondrial research (Review). Int. J. Mol. Med..

[51] Rottenberg H., Hoek J.B. (2017). The path from mitochondrial ROS to aging runs through the mitochondrial permeability transition pore. Aging Cell.

[52] Villalpando-Rodriguez G.E., Gibson S.B. (2021). Reactive Oxygen Species (ROS) Regulates Different Types of Cell Death by Acting as a Rheostat. Oxid. Med. Cell. Longev..

[53] Zhao R.Z., Jiang S., Zhang L., Yu Z.B. (2019). Mitochondrial electron transport chain, ROS generation and uncoupling (Review). Int. J. Mol. Med..

[54] Sousa J.S., D’Imprima E., Vonck J. (2018). Mitochondrial Respiratory Chain Complexes. Subcell. Biochem..

[55] Sies H. (2017). Hydrogen peroxide as a central redox signaling molecule in physiological oxidative stress: Oxidative eustress. Redox Biol..

[56] Niki E. (2016). Oxidative stress and antioxidants: Distress or eustress?. Arch. Biochem. Biophys..

[57] Pizzino G., Irrera N., Cucinotta M., Pallio G., Mannino F., Arcoraci V., Squadrito F., Altavilla D., Bitto A. (2017). Oxidative Stress: Harms and Benefits for Human Health. Oxid. Med. Cell. Longev..

[58] Wang Y., Branicky R., Noë A., Hekimi S. (2018). Superoxide dismutases: Dual roles in controlling ROS damage and regulating ROS signaling. J. Cell Biol..

[59] Brand J.S., Gaillard R., Wes J., McEachan R.R.C., Wright J., Voerman E., Felix J.F., Tilling K., Lawlor D.A. (2019). Associations of maternal quitting, reducing, and continuing smoking during pregnancy with longi-tudinal fetal growth: Findings from Mendelian randomization and parental negative control studies. PLoS Med..

[60] Pereira B., Figueiredo B., Pinto T.M., Míguez M.C. (2020). Effects of Tobacco Consumption and Anxiety or Depression during Pregnancy on Maternal and Neonatal Health. Int. J. Environ. Res. Public Health.

[61] Ioakeimidis N., Vlachopoulos C., Katsi V., Tousoulis D. (2018). Smoking cessation strategies in pregnancy: Current concepts and controversies. Hell. J. Cardiol..

[62] Langley-Evans S.C., Pearce J., Ellis S. (2022). Overweight, obesity and excessive weight gain in pregnancy as risk factors for adverse pregnancy outcomes: A narrative review. J. Hum. Nutr. Diet..

[63] Lewandowska M., Więckowska B., Sajdak S. (2020). Pre-pregnancy obesity, excessive gestational weight gain, and the risk of pregnancy-induced hypertension, and gestational diabetes mellitus. J. Clin. Med..

[64] Zygula A., Kosinski P., Wroczynski P., Makarewicz-Wujec M., Pietrzak B., Wielgos M., Giebultowicz J. (2020). Oxidative Stress Markers Differ in Two Placental Dysfunction Pathologies: Pregnancy-Induced Hypertension and Intrauterine Growth Restriction. Oxid. Med. Cell. Longev..

[65] Chiarello D.I., Abad C., Rojas D., Toledo F., Vázquez C.M., Mate A., Sobrevia L., Marín R. (2018). Oxidative stress: Normal pregnancy versus preeclampsia. Biochim. Biophys. Acta Mol. Basis Dis..

[66] Quan W., Zeng M., Jiao Y., Li Y., Xue C., Liu G., Wang Z., Qin F., He Z., Chen J. (2021). Western Dietary Patterns, Foods, and Risk of Gestational Diabetes Mellitus: A Systematic Review and Meta-Analysis of Pro-spective Cohort Studies. Adv. Nutr..

[67] Osorio-Yáñez C., Gelaye B., Qiu C., Bao W., Cardenas A., Enquobahrie D.A., Williams M.A. (2017). Maternal intake of fried foods and risk of gestational diabetes mellitus. Ann. Epidemiol..

[68] Liang Y., Gong Y., Zhang X., Yang D., Zhao D., Quan L., Zhou R., Bao W., Cheng G. (2018). Dietary Protein Intake, Meat Consumption, and Dairy Consumption in the Year Preceding Pregnancy and During Pregnancy and Their Associations with the Risk of Gestational Diabetes Mellitus: A Prospective Cohort Study in Southwest China. Front. Endocrinol..

[69] Marí-Sanchis A., Díaz-Jurado G., Basterra-Gortari F.J., de la Fuente-Arrillaga C., Martínez-González M.A., Bes-Rastrollo M. (2018). Association between pre-pregnancy consumption of meat, iron intake, and the risk of gestational diabetes: The SUN project. Eur. J. Nutr..

[70] Li S., Tollefsbol T.O. (2021). DNA methylation methods: Global DNA methylation and methylomic analyses. Methods.

[71] Shang L., Huang L., Yang L., Leng L., Qi C., Xie G., Wang R., Guo L., Yang W., Chung M.C. (2021). Impact of air pollution exposure during various periods of pregnancy on term birth weight: A large-sample, retrospective population-based cohort study. Environ. Sci. Pollut. Res..

[72] Shang L., Huang L., Yang W., Qi C., Yang L., Xin J., Wang S., Li D., Wang B., Zeng L. (2019). Maternal exposure to PM2.5 may increase the risk of congenital hypothyroidism in the offspring: A na-tional database based study in China. BMC Public Health.

[73] Smith R.B., Fecht D., Gulliver J., Beevers S.D., Dajnak D., Blangiardo M., Ghosh R.E., Hansell A.L., Kelly F.J., Anderson H.R. (2017). Impact of London’s road traffic air and noise pollution on birth weight: Retrospective population based cohort study. BMJ (Clin. Res. Ed.).

[74] He T., Zhu J., Wang J., Ren X., Cheng G., Liu X., Ma Q., Zhang Y., Li Z., Ba Y. (2018). Ambient air pollution, H19/DMR methylation in cord blood and newborn size: A pilot study in Zhengzhou City, China. Chemosphere.

[75] Lin Y., Zhou L., Xu J., Luo Z., Kan H., Zhang J., Yan C., Zhang J. (2017). The impacts of air pollution on maternal stress during pregnancy. Sci. Rep..

[76] Yang L., Xie G., Yang W., Wang R., Zhang B., Xu M., Sun L., Xu X., Xiang W., Cui X. (2023). Short-term effects of air pollution exposure on the risk of preterm birth in Xi’an, China. Ann. Med..

[77] Zhang Y., Liu S., Wang Y., Wang Y. (2022). Causal relationship between particulate matter 2.5 and hypothy-roidism: A two-sample Mendelian randomization study. Front. Public Health.

[78] Mainprize J.G., Yaffe M.J., Chawla T., Glanc P. (2023). Effects of ionizing radiation exposure during pregnancy. Abdom. Radiol..

[79] Shih B.B., Farrar M.D., Vail A., Allan D., Chao M.R., Hu C.W., Jones G.D.D., Cooke M.S., Rhodes L.E. (2020). In-fluence of skin melanisation and ultraviolet radiation on biomarkers of systemic oxidative stress. Free Radic. Biol. Med..

[80] Di Meo S., Venditti P. (2020). Evolution of the Knowledge of Free Radicals and Other Oxidants. Oxid. Med. Cell. Longev..

[81] Jîtcă G., Ősz B.E., Tero-Vescan A., Miklos A.P., Rusz C.-M., Bătrînu M.-G., Vari C.E. (2022). Positive Aspects of Oxidative Stress at Different Levels of the Human Body: A Review. Antioxidants.

[82] Zhelev Z., Georgieva E., Lazarova D., Semkova S., Aoki I., Gulubova M., Higashi T., Bakalova R. (2019). “Redox Imaging to Distinguish Cells with Different Proliferative Indexes: Superoxide, Hydroperoxides, and Their Ratio as Potential Biomarkers. Oxid. Med. Cell Longev..

[83] Conrad M., Kagan V.E., Bayir H., Pagnussat G.C., Head B., Traber M.G., Stockwell B.R. (2018). Regulation of lipid peroxidation and ferroptosis in diverse species. Genes. Dev..

[84] Nandi A., Yan L.J., Jana C.K., Das N. (2019). Role of Catalase in Oxidative Stress- and Age-Associated Degenerative Diseases. Oxid. Med. Cell Longev..

[85] Thilaganathan B. (2017). Placental syndromes: Getting to the heart of the matter. Ultrasound Obstet. Gynecol..

[86] Beharier O., Tyurin V.A., Goff J.P., Guerrero-Santoro J., Kajiwara K., Chu T., Tyurina Y.Y., Croix C.M.S., Wallace C.T., Parry S. (2020). PLA2G6 guards placental trophoblasts against ferroptotic injury. Proc. Natl. Acad. Sci. USA.

[87] Iuchi K., Takai T., Hisatomi H. (2021). Cell Death via Lipid Peroxidation and Protein Aggregation Diseases. Biology.

[88] Collin F. (2019). Chemical Basis of Reactive Oxygen Species Reactivity and Involvement in Neurodegenerative Diseases. Int. J. Mol. Sci..

[89] Ayala A., Muñoz M.F., Argüelles S. (2014). Lipid peroxidation: Production, metabolism, and signaling mechanisms of malondialdehyde and 4-hydroxy-2-nonenal. Oxid. Med. Cell. Longev..

[90] Iuchi K. (2021). Manipulation of cell fate by fatty acids and oxidized fatty acids. Agric. Biotechnol..

[91] Kowalczyk P., Sulejczak D., Kleczkowska P., Bukowska-Ośko I., Kucia M., Popiel M., Wietrak E., Kramkowski K., Wrzosek K., Kaczyńska K. (2021). Mitochondrial Oxidative Stress—A Causative Factor and Therapeutic Target in Many Diseases. Int. J. Mol. Sci..

[92] Burton G.J., Cindrova-Davies T., Yung H.W., Jauniaux E. (2021). Hypoxia And Reproductive Health: Oxygen and development of the human placenta. Reproduction.

[93] Kelley A.S., Banker M., Goodrich J.M., Dolinoy D.C., Burant C., Domino S.E., Smith Y.R., Song P.X.K., Padmanabhan V. (2019). Early pregnancy exposure to endocrine disrupting chemical mixtures are associated with inflammatory changes in maternal and neonatal circulation. Sci. Rep..

[94] Aleksandrova K., Koelman L., Rodrigues C.E. (2021). Dietary patterns and biomarkers of oxidative stress and inflammation: A systematic review of observational and intervention studies. Redox Biol..

[95] Davis C.R., Bryan J., Hodgson J.M., Woodman R., Murphy K.J. (2017). A Mediterranean Diet Reduces F2-Isoprostanes and Triglycerides among Older Australian Men and Women after 6 Months. J. Nutr..

[96] Morales E., García-Serna A.M., Larqué E., Sánchez-Campillo M., Serrano-Munera A., Martinez-Graciá C., Santaella-Pascual M., Suárez-Martínez C., Vioque J., Noguera-Velasco J.A. (2022). Dietary Patterns in Pregnancy and Biomarkers of Oxidative Stress in Mothers and Offspring: The NELA Birth Cohort. Front. Nutr..

